# Associations of genetically predicted fatty acid levels across the phenome: A mendelian randomisation study

**DOI:** 10.1371/journal.pmed.1004141

**Published:** 2022-12-29

**Authors:** Loukas Zagkos, Marie-Joe Dib, Rui Pinto, Dipender Gill, Fotios Koskeridis, Fotios Drenos, Georgios Markozannes, Paul Elliott, Verena Zuber, Kostas Tsilidis, Abbas Dehghan, Ioanna Tzoulaki

**Affiliations:** 1 Department of Epidemiology and Biostatistics, School of Public Health, Imperial College London, London, United Kingdom; 2 UK Dementia Research Institute, Imperial College London, London, United Kingdom; 3 Chief Scientific Advisor Office, Research and Early Development, Novo Nordisk, Copenhagen, Denmark; 4 Medical Research Council Biostatistics Unit, University of Cambridge, Cambridge, United Kingdom; 5 Department of Hygiene and Epidemiology, University of Ioannina Medical School, Ioannina, Greece; 6 Department of Life Sciences, College of Health, Medicine and Life Sciences, Brunel University London, Uxbridge, United Kingdom; 7 Institute of Cardiovascular Sciences, University College London, London, United Kingdom; 8 BHF Centre of Excellence at Imperial College London, London, United Kingdom; Chinese University of Hong Kong, CHINA

## Abstract

**Background:**

Fatty acids are important dietary factors that have been extensively studied for their implication in health and disease. Evidence from epidemiological studies and randomised controlled trials on their role in cardiovascular, inflammatory, and other diseases remains inconsistent. The objective of this study was to assess whether genetically predicted fatty acid concentrations affect the risk of disease across a wide variety of clinical health outcomes.

**Methods and findings:**

The UK Biobank (UKB) is a large study involving over 500,000 participants aged 40 to 69 years at recruitment from 2006 to 2010. We used summary-level data for 117,143 UKB samples (base dataset), to extract genetic associations of fatty acids, and individual-level data for 322,232 UKB participants (target dataset) to conduct our discovery analysis. We studied potentially causal relationships of circulating fatty acids with 845 clinical diagnoses, using mendelian randomisation (MR) approach, within a phenome-wide association study (PheWAS) framework. Regression models in PheWAS were adjusted for sex, age, and the first 10 genetic principal components. External summary statistics were used for replication. When several fatty acids were associated with a health outcome, multivariable MR and MR-Bayesian method averaging (MR-BMA) was applied to disentangle their causal role. Genetic predisposition to higher docosahexaenoic acid (DHA) was associated with cholelithiasis and cholecystitis (odds ratio per mmol/L: 0.76, 95% confidence interval: 0.66 to 0.87). This was supported in replication analysis (FinnGen study) and by the genetically predicted omega-3 fatty acids analyses. Genetically predicted linoleic acid (LA), omega-6, polyunsaturated fatty acids (PUFAs), and total fatty acids (total FAs) showed positive associations with cardiovascular outcomes with support from replication analysis. Finally, higher genetically predicted levels of DHA (0.83, 0.73 to 0.95) and omega-3 (0.83, 0.75 to 0.92) were found to have a protective effect on obesity, which was supported using body mass index (BMI) in the GIANT consortium as replication analysis. Multivariable MR analysis suggested a direct detrimental effect of LA (1.64, 1.07 to 2.50) and omega-6 fatty acids (1.81, 1.06 to 3.09) on coronary heart disease (CHD). MR-BMA prioritised LA and omega-6 fatty acids as the top risk factors for CHD. Although we present a range of sensitivity analyses to the address MR assumptions, horizontal pleiotropy may still bias the reported associations and further evaluation in clinical trials is needed.

**Conclusions:**

Our study suggests potentially protective effects of circulating DHA and omega-3 concentrations on cholelithiasis and cholecystitis and on obesity, highlighting the need to further assess them as prevention treatments in clinical trials. Moreover, our findings do not support the supplementation of unsaturated fatty acids for cardiovascular disease prevention.

## Abstract 2

Loukas Zagkos and colleagues conduct Mendelian randomisation analysis to investigate potential causal associations of circulating fatty acids and 845 disease outcomes in the UK Biobank.

## Author summary

**Why was this study done?:**

- Epidemiological studies have investigated the influence of dietary fatty acids on several chronic diseases including cardiovascular disease (CVD), inflammatory diseases, cancers, and neurodegenerative conditions.
- The evidence is conflicting and inconclusive and is not supported by randomised controlled trials (RCTs) on fatty acids supplementation.
- The fact that the human fatty acid metabolome partly reflects the fatty acid intake was leveraged in this work.

**What did the researchers do and find?:**

- Mendelian randomisation analysis was conducted to investigate potential causal associations of circulating fatty acids and 845 disease outcomes in UK Biobank.
- We found that 1 mmol/L increase in genetically predicted docosahexaenoic acid (DHA) was associated with 24% reduced odds of cholelithiasis and cholecystitis, as well as 17% reduced odds of obesity.
- One mmol/L higher genetically predicted linoleic acid (LA) was associated with 27% increased odds of ischemic heart disease.
- When assessed with other fatty acid classes simultaneously, 1 mmol/L higher genetically predicted LA and omega-6 fatty acids were associated with higher odds of coronary heart disease (64% and 81% increase, respectively).

**What do these findings mean?:**

- Our results support the prioritisation of RCTs examining the role of primary and secondary prevention of cholelithiasis via dietary modification or supplementation of DHA and other omega-3 fatty acids.
- Our principal findings also provide evidence against the supplemental use of fatty acids for CVD prevention.
- Triangulation of evidence for causal inference of fatty acid levels through experimental studies susceptible to different assumptions is needed to strengthen the evidence.

## Introduction

Fatty acids (FAs) are major constituents of several lipid species and are involved in diverse metabolic pathways and biochemical processes in normal cells [1]. Their role in health and disease has been extensively studied; however, the mechanisms by which fatty acids may exert their effects on health outcomes are still not fully understood, and the causality between different fatty acids and health outcomes has not been established. Numerous epidemiological studies have investigated the quantity and quality of dietary fatty acids in relation to several chronic diseases including cardiovascular disease (CVD), cancers, neurodegenerative conditions, and inflammatory diseases [2,3]. However, this evidence remains inconclusive [4], and it is often not supported by randomised controlled trials (RCTs) on fatty acid supplementation, which have also shown contradictory results [5–8]. There is, therefore, an urgent need to establish the potential causal role of fatty acids in relation to a wide range of phenotypes as fatty acid supplements are the most commonly consumed nonvitamin/nonmineral dietary supplements by both adults and children in western societies [9,10].

The human fatty acid metabolome partly reflects the dietary fatty acid intake and has the potential to explain their pathophysiological roles. Circulating fatty acid levels are influenced by environmental and genetic factors [11–15] and can be used as biomarkers of fatty acid intake. The present study aimed to assess potentially causal associations between circulating fatty acid levels and the risk of different health outcomes using mendelian randomisation (MR) analysis. MR analysis uses genetic polymorphisms that are randomised by nature and can overcome the limitations of traditional epidemiological studies, as the random allocation of alleles during conception minimises confounding, and their presence from birth prevents reverse causation. Our study leveraged recent genetic data generated from over 115,000 UK Biobank (UKB) participants on circulating levels of 8 nuclear magnetic resonance (NMR) fatty acid measures, providing unprecedented power for fatty acids MR studies. Specifically, we investigated associations for docosahexaenoic acid (DHA) and omega-3 fatty acids, linoleic acid (LA), and omega-6 fatty acids, as well as the fatty acid classes: monounsaturated fatty acids (MUFAs), polyunsaturated fatty acids (PUFAs), saturated fatty acids (SFAs), and total fatty acids (total FAs). We implemented a systematic approach to examine causal pathways between each fatty acid trait and the human phenome, followed by multivariable MR and the MR-Bayesian model averaging (MR-BMA) framework to identify direct effects of different fatty acids and to select the ones that are the most likely causal risk factors for disease phenotypes.

## Methods

### Study design

**Fig 1:**
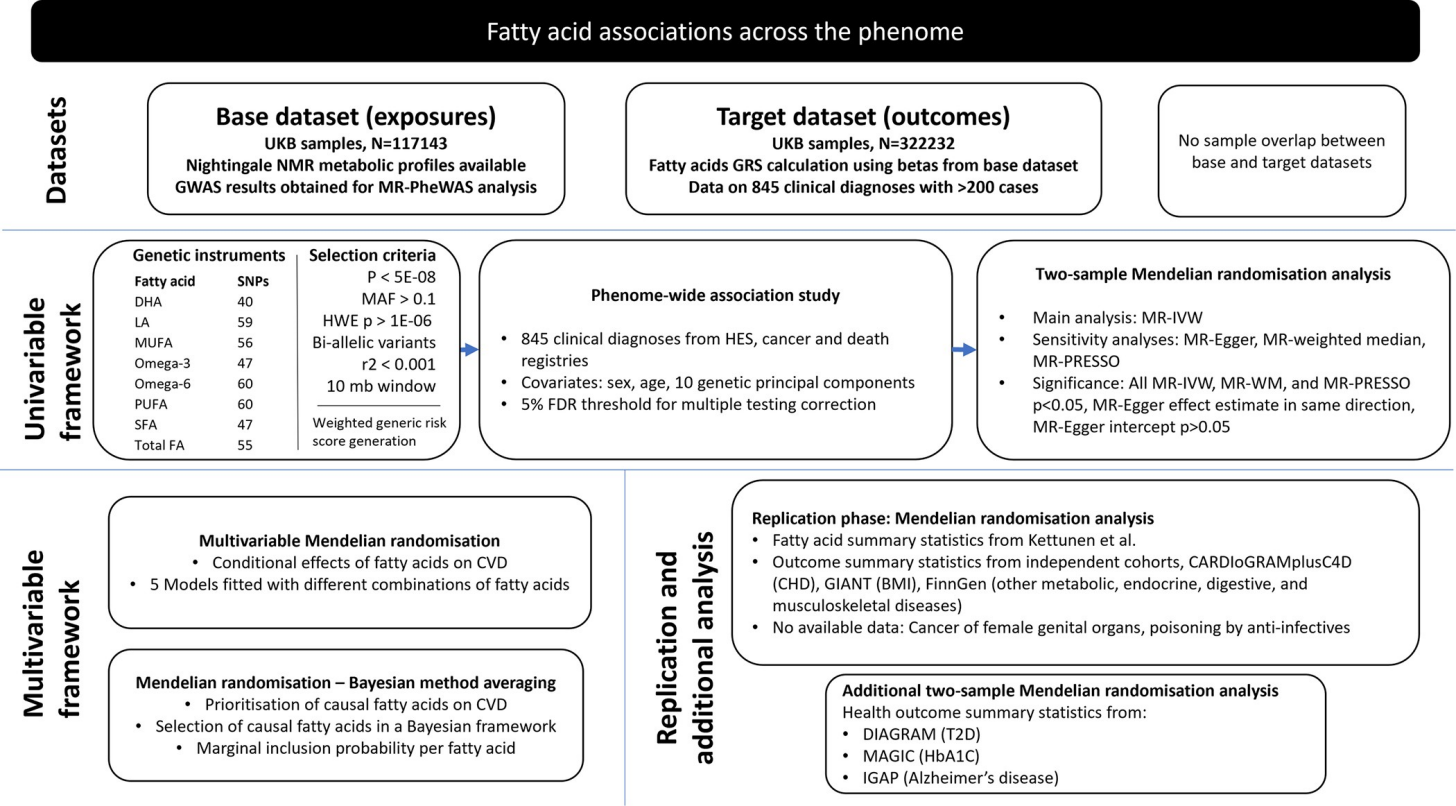
Overview of the study. BMI, body mass index; CHD, coronary heart disease; CVD, cardiovascular disease; DHA, docosahexaenoic acid; FDR, false discovery rate; GRS, genetic risk score; GWAS, genome-wide association study; HbA1c, haemoglobin A1c; HES, Hospital Episode Statistics; HWE p, Hardy–Weinberg equilibrium *p*-value; IVW, inverse-variance weighted; LA, linoleic acid; MAF, minor allele frequency; MR-PheWAS, mendelian randomisation–phenome-wide association study; MUFA, monounsaturated fatty acid; NMR, nuclear magnetic resonance; *P*, *p*-value; PUFA, polyunsaturated fatty acid; SFA, saturated fatty acid; total FA, total fatty acid; T2D, type 2 diabetes.

The study design is shown in **Fig 1**. This study used a phenome-wide association study (PheWAS) to identify associations between genetically predicted fatty acid concentrations and several diseases in UKB. For statistically significant associations, a two-sample MR of summary-level data was conducted to further investigate the causal role of fatty acids on health outcomes followed by multivariable MR and MR-BMA. We further attempted to replicate statistically significant MR associations using alternative data sources for the exposures (fatty acids) and the outcomes. This study is reported as per the Strengthening the Reporting of Observational Studies in Epidemiology (STROBE) guideline, specific for MR (S1 Checklist).

### Data sources

#### UK Biobank

The UKB [16] is a large ongoing prospective study involving over 500,000 participants aged 40 to 69 years at recruitment from 2006 to 2010. The study has collected biological samples and a wide range of phenotypic data from its participants, including data from questionnaires, physical measures, sample assays, genome-wide genotyping, and longitudinal follow-up for a plethora of health-related outcomes. Details of genotyping and data management have been described [17].

Recently, UKB released high-throughput NMR metabolic biomarkers for a random subset of 117,994 participants at baseline assessment including measurements of 8 circulating fatty acids: DHA, LA, MUFAs, PUFAs, omega-3, omega-6, SFAs, and total FAs.

#### Fatty acids genetic instruments

We obtained genetic summary-level data from genome-wide association studies (GWAS) for the 8 measured fatty acids on 114,999 European UKB participants from the MRC IEU OpenGWAS project [18]. Single nucleotide polymorphisms (SNPs) with a minor allele frequency (MAF) greater than 0.1 and a Hardy–Weinberg equilibrium (HWE) *P* value greater than 10^−6^ were considered. Multiallelic SNPs were excluded from the analysis. Independent SNPs associated with each of the 8 fatty acid concentrations were identified at a genome-wide significance threshold (*P* < 5 × 10^−8^). Independence of SNPs was assessed through clumping for linkage disequilibrium (LD) r^2^ < 0.001 using a window of 10 million bases and the 1,000 genomes phase 3 European reference panel through the open-source association toolset plink [19]. In the replication phase, we used the Kettunen and colleagues’ GWAS [20] as an alternative source of beta estimates for genetic variants of the same fatty acids using the same criteria as above. To rule out exogenous factors that may affect the genetic associations with the fatty acids, we excluded 13,577 omega-3 supplementation and/or lipid lowering drug users with available metabolic profiles in UKB, recalculated the fatty acid genetic associations, and monitored the percentage change in genetic association estimates.

#### Health outcomes

For the PheWAS analysis, individual-level data from UKB were used for clinical health outcomes with more than 200 cases, obtained from national registries, such as Hospital Episode Statistics (HES), cancer registry, and death registry. Cases were defined according to ICD-9 and ICD-10 codes (International Classification of Diseases, ninth and 10th revisions). We also used alternative (independent) GWAS sources for the outcomes, which showed statistically significant associations in the two-sample MR analyses. We selected the largest published GWAS for each outcome, and when there was no available GWAS, we used FinnGen (https://r5.finngen.fi/) estimates. In detail, we used GIANT consortium for body mass index (BMI) [21], CARDIoGRAMplusC4D for coronary heart disease (CHD) [22], IGAP [23] for Alzheimer’s disease, MAGIC [24] for HbA1c and DIAGRAM [25] for type 2 diabetes (T2D), and FinnGen (https://r5.finngen.fi/) for cholelithiasis and “other biliary tract disease”.

### Statistical analysis

#### Genetic risk score generation

A weighted genetic risk score (GRS) was generated for each fatty acid concentration for UKB participants by adding up the number of risk-increasing alleles for all the variants selected as instruments. The number of risk-increasing alleles at each SNP was multiplied by the effect estimate of the association of that SNP with the exposure. This analysis was performed in the “target” UKB dataset, which excludes individuals with available NMR metabolic biomarker data (base dataset), so as to avoid sample overlap between the base dataset (the 117,143 UKB samples used for the estimation of the effect of SNPs on the exposures) and the target dataset (the 322,232 UKB samples used for the generation of the GRS, after excluding the base dataset). For the GRS generation in the target dataset, we excluded non-European participants, one randomly selected participant from each pair of up to third-degree relatives (kinship coefficient > 0.0884) [17] and individuals with discordant reported sex and genetic sex.

#### Phenome-wide association study

A PheWAS aims to investigate possible associations between a single GRS or genotype and multiple phenotypes. We conducted 8 PheWAS to investigate potential associations between the GRS of each specific fatty acid measurement and 845 clinical diagnoses. All regression models were adjusted for sex, age, and the first 10 genetic principal components, estimated by UKB (Data-Field 22009). To account for type 1 error inflation due to multiple testing, we used the false discovery rate (FDR) method, controlled at 5% significance level by the Benjamini–Hochberg procedure [26].

#### Two-sample mendelian randomisation and sensitivity analyses

For the associations reaching 5% FDR in the GRS-PheWAS analysis, we performed two-sample MR analysis. MR assesses causal relationships using genetic variants as instrumental variables. A genetic variant can be considered as an instrumental variable if it satisfies three assumptions: it is robustly associated with the exposure, it is independent of any confounders of the exposure–outcome relationship, and it is associated with the outcome only via the exposure. To obtain the effect estimates of the associations between the genetic instruments and the health outcomes, we fitted logistic regression models in UKB, adjusting for sex, age, and the first 10 genetic principal component. In this MR framework, there was no sample overlap between the UKB populations used for the fatty acids (base dataset) and for the clinical diagnoses (target dataset). The main analysis was conducted using the random-effects inverse-variance weighted (IVW) method, which provides precise causal estimates, assuming that all variants are valid instrumental variables [27]. In sensitivity analyses, we applied the MR-Egger method to explore the assumption of no pleiotropy in genetic instruments [28]. In addition, we performed the MR-weighted median method, which returns an accurate causal estimate, provided that at least 50% of the weight in the analysis comes from valid instrumental variables [29]. The I^2^ statistic was calculated to detect heterogeneity among the MR estimates obtained from multiple genetic variants. Last, we used MR-PRESSO, which detects and removes pleiotropic variants based on their contributions to heterogeneity [30]. We considered associations to be statistically significant if *P* values in MR-IVW, MR-weighted median, and MR-PRESSO methods were smaller than 0.05, with supportive MR-Egger. MR-Egger was considered supportive when the effect estimate was in the same direction as MR-IVW and the MR-Egger intercept was statistically nonsignificant (*P* > 0.05). The F-statistic for each genetic instrument was generated to assess their strength. We included instruments with F-statistic > 10 in the analysis. Moreover, as a sensitivity analysis for the fatty acid associations with CHD, BMI, and cholelithiasis, we excluded the genetic instruments within 500 kb of the FADS locus (chromosome 11: 61,067,097 to 62,134,826).

To assess the robustness of our findings, we attempted to replicate findings using alternative data sources for the exposure and the outcome, respectively. We performed the same sequence of MR analyses (main and sensitivity analyses described above) using Kettunen and colleagues’ GWAS [20] for fatty acids and the largest available published GWAS for each outcome of interest (see Health outcomes above).

All estimates were reported as odds ratio (OR) per 1 mmol/L increase in fatty acid concentrations, together with their 95% confidence interval.

#### Multivariable mendelian randomisation

To disentangle the relationships between correlated circulating fatty acids and CHD, we conducted two analytical approaches. Firstly, we conducted multivariable MR, a statistical method for estimation of causal effects using genetic variants associated with more than one risk factor [31]. Multivariable MR allows an estimation of the direct effect of each fatty acid on CHD, that is, an effect that is not mediated by any other fatty acid in the model. Secondly, we conducted MR-BMA, an extension of multivariable MR, which selects and prioritises candidate highly correlated risk factors with shared genetic predictors using a Bayesian framework as previously described [32]. For MR-BMA, we considered each set of fatty acids in turn, and for each set of fatty acids, we undertook a multivariable MR analysis using weighted regression based on summarised genetic data. Regression models were assessed using goodness of fit, and a score was assigned to each set of fatty acids that represented the model’s posterior probability of that set being the true causal risk factors. Then, for each of the candidate fatty acids, we summed up the posterior probability over models including that fatty acid to compute its marginal inclusion probability, representing its probability of being a causal determinant of disease risk. We also calculated the model-averaged causal effect, which represents the average causal effect across models including that fatty acid. For all instruments, we generated the conditional F-statistic to assess their strength [33]. Using both approaches, we fitted five models based on the hierarchical order of the fatty acid measurements, considering different combinations to avoid overlap between fatty acids (e.g., DHA and omega-3). We fitted a model with DHA, LA, MUFA, and SFA; a second model considering omega-3, omega-6, MUFA, and SFA; and a third with PUFA, MUFA, and SFA. Additionally, we assessed the independent effects of PUFAs alone on CHD, i.e., DHA and LA (model 4), and omega-3 with omega-6 (model 5).

#### Statistical software

Analysis was conducted in R version 4.0.2 [34], phenome-wide associations were conducted using R package “PheWAS” [35], two-sample MR was performed using “TwoSampleMR” [36] and “MRPRESSO” [30] R packages, and MR-BMA was conducted using the methodology provided in [32]. Figures were produced using the R package “forestplot” [37].

## Results

Fatty acid GRS was calculated on UKB participants, after excluding those with available NMR metabolic biomarker data, to avoid sample overlap between the base dataset (the 117,143 UKB samples used for the estimation of the effect of SNPs on the exposures) and the target dataset (the 322,232 UKB samples used for the generation of the GRS, after excluding the base dataset).

**Fig 2:**
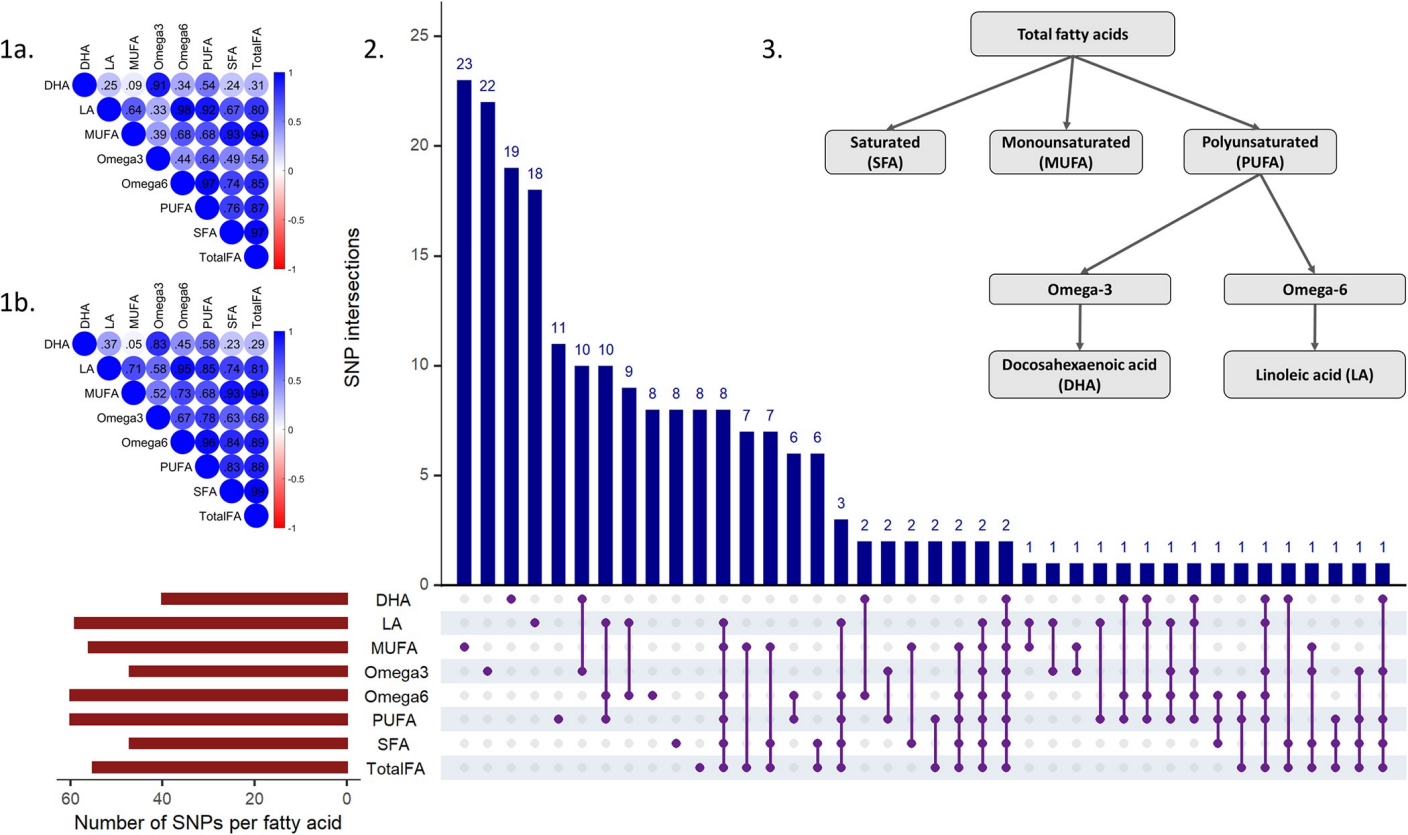
**(1a)** Pearson correlation coefficient heatmap between phenotypic circulating fatty acid measurements. **(1b)** Genetic correlation heatmap between the fatty acid measurements. **(2)** Additional information on the genetic instruments used in the analysis. Independent biallelic genetic variants that are associated with fatty acids below *P* = 5 × 10^−8^, have MAF > 0.1 and HWE *P* > 10^−6^ were used as instruments. Independence was assessed through clumping with r^2^ < 0.001 and 10 million bases clumping window. Dark red horizontal bars at the left bottom corner show the total number of genetic variants used as instruments per fatty acid. Dark blue vertical bars depict the number of genetic variants within each intersection. SNP intersections are defined with the purple dots below the vertical bars, indicating SNP overlap between groups, e.g., the first bar refers to MUFA having 23 unique SNPs, the sixth bar refers to 10 common SNPs between DHA and omega-3 etc. (**3)** Hierarchical order of the fatty acids. DHA, docosahexaenoic acid; HWE, Hardy–Weinberg equilibrium; LA, linoleic acid; MAF, minor allele frequency; MUFA, monounsaturated fatty acid; PUFA, polyunsaturated fatty acid; SFA, saturated fatty acid; SNP, single nucleotide polymorphism; total FA, total fatty acid.

Descriptive characteristics of the 117,143 UKB samples (base dataset) and 322,232 UKB samples (target dataset) used in the discovery phase and the cohorts used in the replication phase are provided in **S1 Table**. Aside from the phenotypic correlations between DHA and LA (Pearson’s R = 0.09), the remaining correlations between fatty acid measurements were relatively high (highest correlation 0.97 between total FA and SFA). Genetic correlations between the fatty acid measurements were estimated using linkage disequilibrium score regression (LDSC) [38] (**S2 Table**) and showed a similar pattern as the phenotypes. As expected, the genetic instruments for each fatty acid shared a number of SNPs, especially those belonging in nested groups, e.g., DHA and omega-3. The hierarchical order of the fatty acid measurements, phenotypic and genetic correlations between the fatty acids and the genetic variants used as instruments in the analysis are provided in **Fig 2**. The list of independent genetic variants used as instrumental variables in this analysis and their corresponding F-statistic are provided in **S3** and **S4** Tables.

### Phenome-wide association study

**Fig 3:**
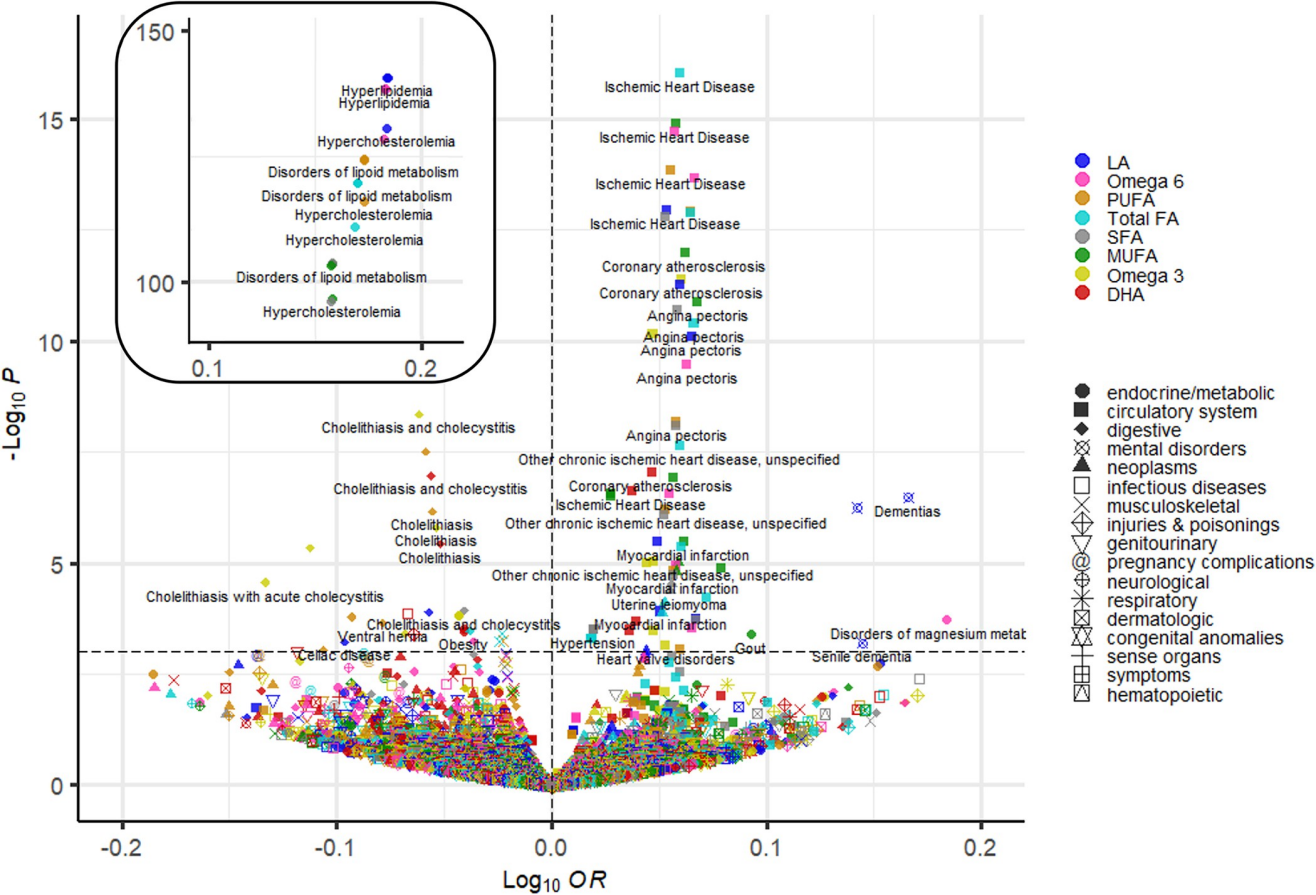
PheWAS results for the associations between 8 weighted GRS of different fatty acids and 845 clinical outcomes in UKB. log(OR) regression estimates are plotted against −log(*P* value). The dashed line represents the 5% FDR threshold, which corresponds to *P* = 9.2 × 10^−4^. Due to their high *P* values, associations with disorders of lipid metabolism, hypercholesterolemia, and hyperlipidaemia can be seen in the top left box. DHA, docosahexaenoic acid; FDR, false discovery rate; GRS, genetic risk score; LA, linoleic acid; MUFA, monounsaturated fatty acid; PheWAS, phenome-wide association study; PUFA, polyunsaturated fatty acid; SFA, saturated fatty acid; total FA, total fatty acid; UKB, UK Biobank.

PheWAS examined 845 distinct clinical diagnoses grouped into 17 disease categories (median number of cases: 879 [range: 200 to 47,166]) (**S5 Table)**. Thirty-five unique clinical outcomes were associated with at least one fatty acid GRS at 5% FDR threshold (*P* = 9.2 × 10^−4^); 9 of these outcomes were within the CVD group (positive associations) and 5 within the digestive group (negative associations) (**Fig 3**; see full results in **S6–S13** Tables and **S1–S8** Figs).

### Mendelian randomisation

MR results are summarised in **S14 Table**. Out of 114 MR tests, 60 associations were statistically significant, following the criteria described in the Methods section. Phenotypes with supporting evidence of causality are discussed below.

**Fig 4:**
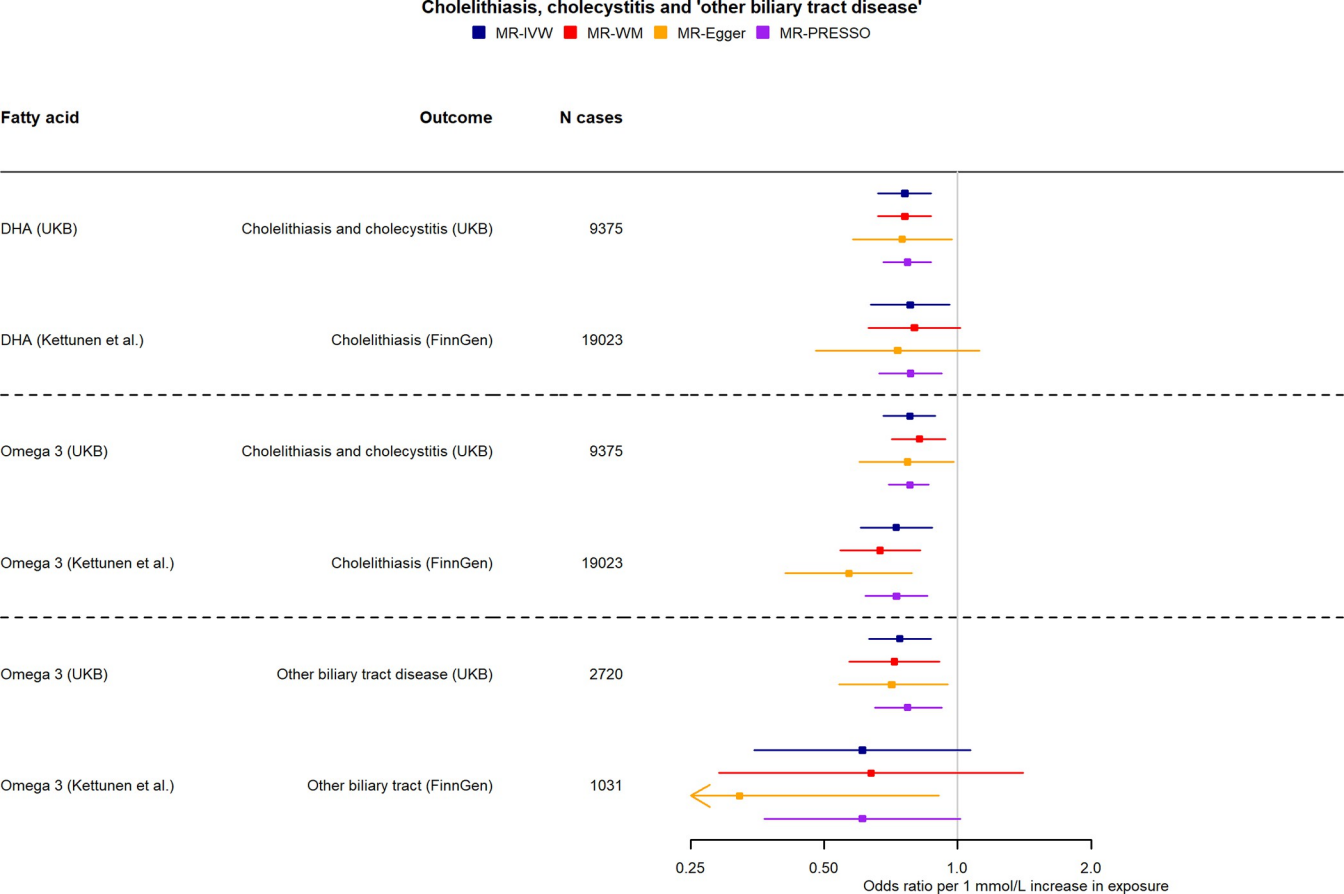
Two-sample MR effect estimates per 1 mmol/L higher fatty acids for cholelithiasis, cholecystitis, and “other biliary tract disease” in (a) using betas from UKB and (b) using betas from Kettunen and colleagues [20] for fatty acids and FinnGen (https://r5.finngen.fi/) for cholelithiasis. Only associations between genetically predicted fatty acids and biliary tract health outcomes with statistically significant (*P* < 0.05) IVW MR estimates in UKB are shown. DHA, docosahexaenoic acid; IVW, inverse-variance weighted; MR, mendelian randomisation; UKB, UK Biobank; WM, weighted-median.

Higher genetically predicted levels of DHA were found to have a protective effect on cholelithiasis and cholecystitis (OR per 1 mmol/L increase in DHA: 0.76, 95% CI: 0.66 to 0.87) and cholelithiasis (0.77, 0.67 to 0.89) **(Fig 4)**. These were supported in replication analysis (FinnGen study as the outcome GWAS) and by the omega-3 FA analyses and were consistent after SNPs in the pleiotropic FADS region were excluded (**S15** and **S16** Tables).

**Fig 5:**
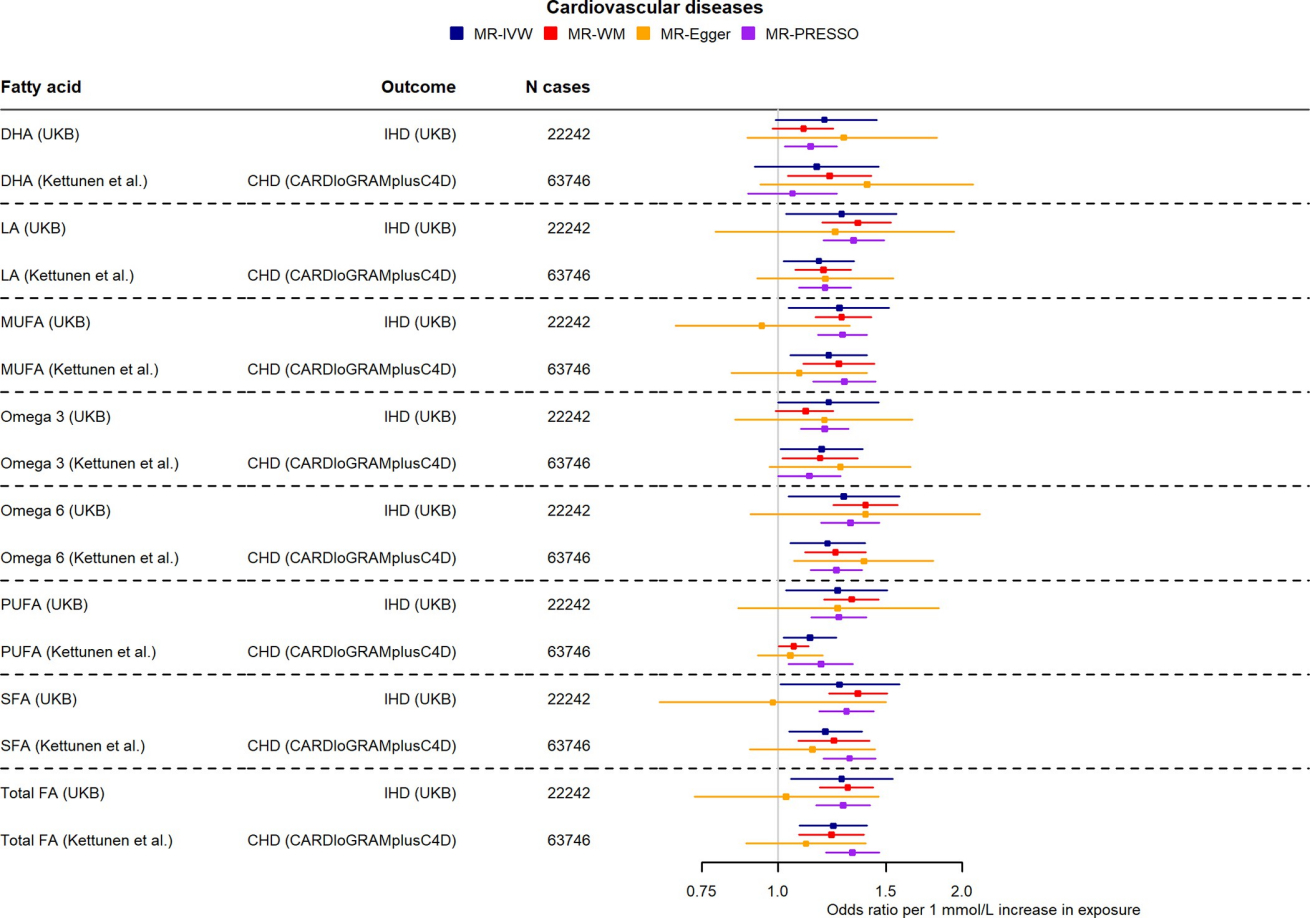
Two-sample MR effect estimates per 1 mmol/L higher fatty acids for different CVD outcomes in (a) UKB (angina pectoris, coronary atherosclerosis, ischemic heart disease) and (b) using Kettunen and colleagues’ [20] betas for fatty acids and CARDIOGRAMplusC4D [22] for ischemic heart disease. Estimates of all examined fatty acids are shown for comparison. CVD, cardiovascular disease; DHA, docosahexaenoic acid; LA, linoleic acid; MUFA, monounsaturated fatty acid; PUFA, polyunsaturated fatty acid; SFA, saturated fatty acid; total FA, total fatty acid; UKB, UK Biobank; WM, weighted-median.

Genetically predicted LA, omega-6, PUFA, and total FA showed positive associations with cardiovascular outcomes. More specifically, we identified an increased risk on ischemic heart disease for higher total FA (1.27, 1.05 to 1.54), PUFA (1.25, 1.03 to 1.51), omega-6 (1.28, 1.04 to 1.58), and LA (1.27, 1.03 to 1.56) per 1 mmol/L increase in genetically predicted levels, as seen in **Fig 5**. These associations were consistent in replication analysis using CARDIoGRAMplusC4D as the outcome GWAS source (**S17 Table**). After excluding SNPs in the FADS locus, all fatty acids retained their significant detrimental effects on CHD, except for DHA and omega-3 (**S16 Table**). Considerable heterogeneity was observed in these analyses (I^2^ statistic > 50%), which, when reduced by removing outliers from MR-PRESSO, resulted in effect estimates supporting a causal effect. Replication analysis using Kettunen and colleagues’ betas for the fatty acids further supported the positive association for genetically predicted LA, omega-6 fatty acids, and PUFA in relation to CHD (**S15 Table**).

**Fig 6:**
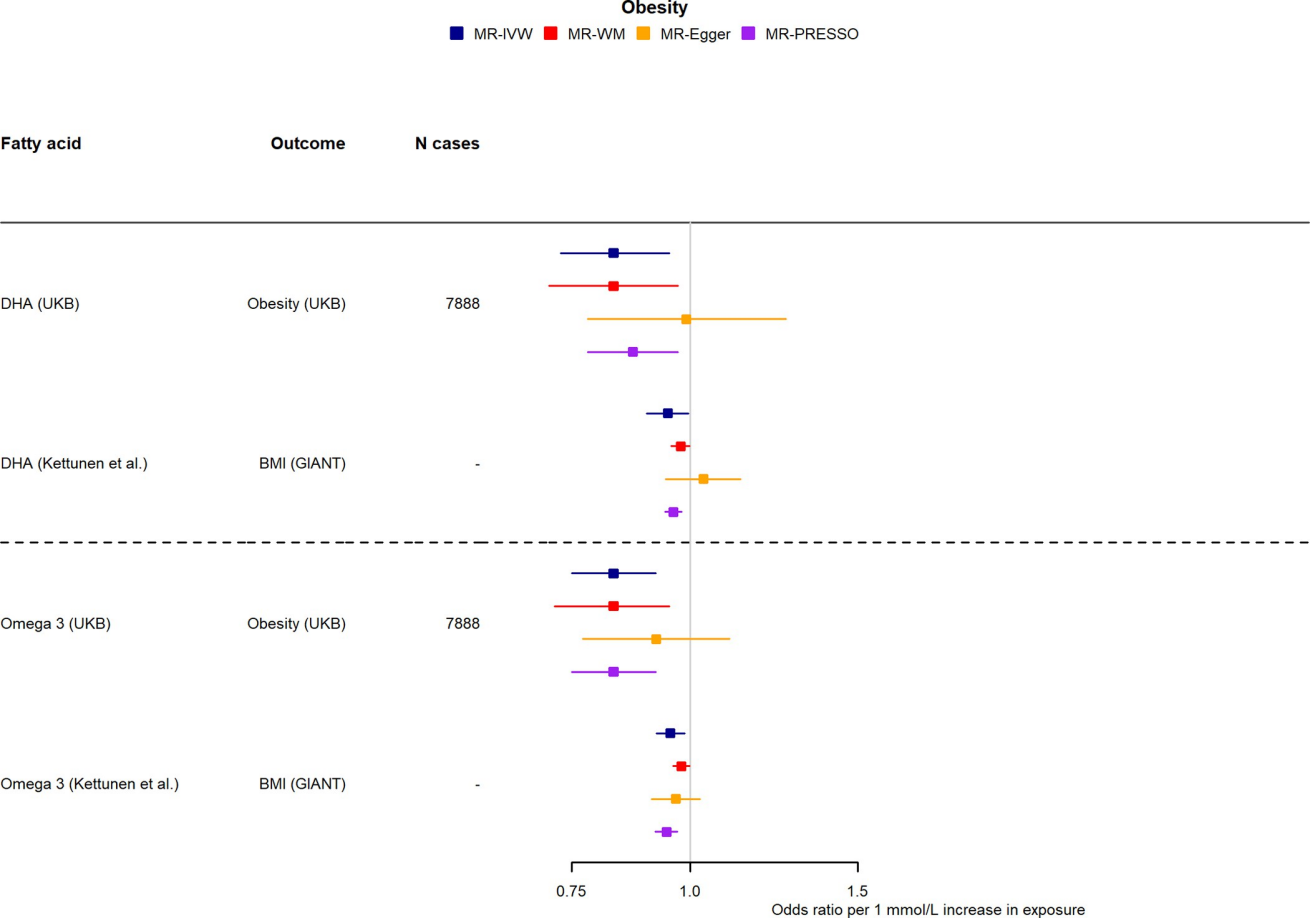
Two-sample MR effect estimates per 1 mmol/L higher fatty acids for obesity in (a) UKB and (b) using Kettunen and colleagues’ [20] betas for fatty acids and betas from GIANT [21] for BMI. Only associations with statistically significant (*P* < 0.05) IVW MR estimates in the discovery phase are shown. BMI, body mass index; DHA, docosahexaenoic acid; IVW, inverse-variance weighted; MR, mendelian randomisation; MUFA, monounsaturated fatty acid; total FA, total fatty acid; UKB, UK Biobank; WM, weighted-median.

Higher genetically predicted levels of DHA and omega-3 were found to have a protective effect on obesity (0.83, 0.73 to 0.95) and (0.83, 0.75 to 0.92), respectively (**Fig 6**). This was further supported in replication analysis using BMI as the outcome of interest (GIANT consortium) (**S15 Table**). These associations with BMI were attenuated to the null when excluding SNPs in the FADS locus (**S16 Table**). Moreover, higher levels of genetically predicted total FA concentration had a protective effect on osteoarthrosis (1.27, 1.02 to 1.57), which was not supported in replication analysis using osteoarthritis as the outcome of interest in FinnGen.

### Multivariable mendelian randomisation

**Fig 7:**
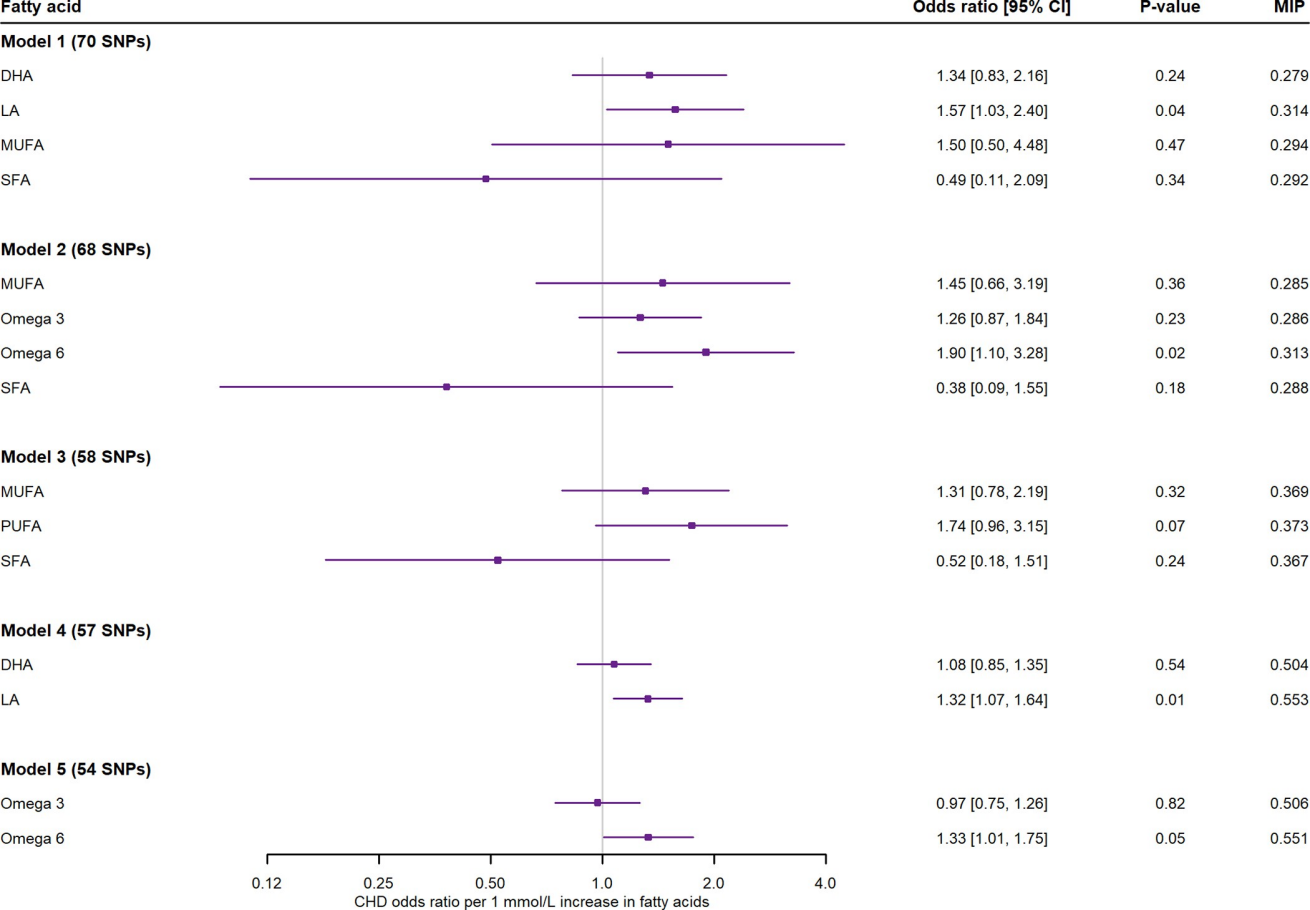
Two-sample multivariable MR effect estimates per 1 mmol/L higher fatty acids on CHD, using UKB betas for fatty acids and betas from CARDIOGRAMplusC4D [22] for CHD. The last column illustrated the MIP per fatty acid (MR-BMA output) to prioritise risk factors on CHD. Five models were fitted to investigate the direct effects of fatty acids. Model 1 considered the simultaneous effects of DHA, LA, MUFA, and SFA; Model 2 the effects of omega-3, omega-6, MUFA, and SFA; Model 3 the effects of PUFA, MUFA, and SFA; Model 4: DHA and LA; and Model 5: omega-3 and omega-6. MIP in the last column. CHD, coronary heart disease; DHA, docosahexaenoic acid; LA, linoleic acid; MIP, marginal inclusion probability; MR, mendelian randomisation; MR-BMA, MR-Bayesian method averaging; MUFA, monounsaturated fatty acid; PUFA, polyunsaturated fatty acid; SFA, saturated fatty acid; UKB, UK Biobank.

Five multivariable models were fitted to examine the direct effects of fatty acids on CHD, according to their hierarchical order (**S18 Table**). Increasing LA retained a potentially causal relationship with CHD when assessed together with DHA, MUFA, and SFA (1.64, 1.07 to 2.50) and when assessed with DHA only (1.32, 1.07 to 1.64). Higher omega-6 was also associated with increased risk of CHD, when omega-3, omega-6, MUFA, and SFA were simultaneously fitted into a model (1.81, 1.06 to 3.09) and when fitted into a model with omega-3 only (1.33, 1.01 to 1.75) (**Fig 7**). In the MR-BMA analysis, LA was narrowly the top-ranked risk factor for CHD in the model with DHA, MUFA, and SFA (marginal inclusion probability, 0.31) and when assessed with DHA (marginal inclusion probability, 0.55), while omega-6 was identified as the prioritised risk factor for CHD (marginal inclusion probability, 0.31) when assessed with omega-3, MUFA, and SFA and (marginal inclusion probability, 0.55) in the model with omega-6.

## Discussion

In this study, we used MR to explore and compare the relationships of fatty acids levels with a wide range of clinical phenotypes across the phenome. Our principal findings include the positive associations between several genetically predicted fatty acids and CVD outcomes with multivariable analysis highlighting omega-6 fatty acids and LA in particular, having an independent causal positive effect on CHD. This adds further evidence against the supplemental use of fatty acids for CVD prevention. Our results also indicate negative associations of higher DHA and omega-3 fatty acid levels with lower risk of cholelithiasis and obesity.

Extensive MR analyses supported a protective causal effect of omega-3, and its main bioactive component DHA, on the combined endpoint of cholelithiasis and cholecystitis with replication in independent databases. Several lines of evidence further support this observation. Cholelithiasis involves the presence of gallstones the vast majority of which are cholesterol stones and primarily composed of cholesterol. Long-term intake of *cis*-unsaturated and monounsaturated fats has been associated with lower risk of gallstone formation in observational settings [39], while in patients with gallstones, omega-3 fatty acid supplementation has been shown to reduce the cholesterol saturation index in bile [40]. Omega-3 also prevented gallstone formation in mice being on a lithogenic diet by increasing the levels of bile phospholipids and suppressing bile mucin formation [41]. Interestingly, analyses did not support causal associations between omega-6 fatty acid levels and cholelithiasis, suggesting that the protective effect of PUFA on the dissolution of bile disorders may occurs mainly via DHA. Overall, these results support the prioritisation of RCTs examining the role of primary and secondary prevention of cholelithiasis via dietary modification or supplementation of DHA and other omega-3 PUFAs.

A protective potentially causal effect of genetically predicted omega-3 fatty acids and especially DHA on obesity was also observed. Several animal and human studies have suggested an association of omega-3 fatty acids on lower adiposity through their inverse association with inflammatory cytokines and leptin [42]. However, the evidence on the beneficial effects of omega-3 supplementation in weight loss or body fat mass in RCTs has, to date, been inconsistent [43]. Our findings support the contribution of omega-3 and DHA in healthy weight status and prevention of obesity compared to other fatty acids; however, further evidence on the actual dose effects and the heterogeneity of effects across different fatty acids is needed to validate these observations.

There has been considerable debate on the higher PUFA levels potentially reducing CVD risk. Omega-3 supplements are widely used for CVD prevention despite lack of support from regulatory bodies at least for secondary prevention [44,45]. A large body of observational studies has supported an association between omega-3—mainly eicosapentaenoic acid (EPA) and DHA—and cardiometabolic diseases, such as ischemic heart disease and T2D [46–49]. However, several RCTs have failed to show an indisputable benefit of omega-3 supplementation on a range of CVD outcomes, while there is some suggestive evidence for a protective effect of high-dose supplementation, which needs replication and further evaluation [4,50]. The present MR findings further augment the conflicting evidence base. In agreement with previous work [51], we found no evidence of protective effect between genetically predicted omega-3 fatty acids and CHD. Our results provided evidence for a possible causal detrimental effect of LA and omega-6 levels in relation to CHD and associated CVD outcomes. These associations remained significant after excluding SNPs in the FADS locus; however, horizontal pleiotropy could still explain these associations. Multivariable MR and MR-BMA analyses indicated LA as having an independent effect on CVD and as the risk factor with the strongest overall evidence for positive association with CHD. Evidence from observational studies on dietary omega-6 fatty acids supports reverse associations with incident CVD [52], while evidence from RCTs showed null associations [53]. The apparent controversy between different lines of evidence may, to a certain extent, be explained by the different definitions of fatty acids between traditional observational studies (mainly dietary fatty acids), RCTs (fatty acid supplementation), and MR (genetically predicted fatty acid levels). Additionally, MR represents the lifetime effects of the genetic variants rather than a short period of intervention usually examined in RCTs. Nonetheless, the present evidence highlighted the need for further research on the effects of different fatty acids on CVD and does not support the otherwise wide use of fatty acid supplements for CVD prevention.

### Strengths and limitations

Our study has notable strengths. First, it benefits from the largest available population data, which allowed us to assess potential sources of heterogeneity in MR findings and to replicate our results in external databases increasing the generalizability and validity of our findings. Additionally, we examined potential beneficial and detrimental effects of fatty acids across the phenome, and we examined 8 different fatty acid categories with multivariable analyses to establish their relative importance extending previous work on PUFA and cardiovascular outcomes [52]. However, our results must be interpreted in the context of a few limitations. Our work was confined to individuals of European descent, thus limiting the amenability of our findings to other ethnic groups. Also, we were unable to assess the effect of fatty acid ratios such as omega-6-to-omega-3 fatty acid that might be important for some of the examined clinical outcomes. Although our study strengthens the case for a causal relationship between fatty acids and several diseases, we cannot prove causality, as this is an exploratory hypothesis generating study that requires further validation in the form of a clinical study. For example, pleiotropy is always a potential problem in MR and occurs when a genetic instrument is associated with the outcome through pathways not including the exposure of interest. We used extensive sensitivity analyses to account for pleiotropy and implemented a Bayesian multivariable approach to select among the studied fatty acids the one more likely to be causal. This is important as the examined fatty acids have high genetic correlation. Therefore, the observed associations could reflect an association between a fatty acid other than those we have investigated, or even a combination of fatty acids. Lastly, exogenous factors may affect the genetic associations with the fatty acids, since 13,577 participants with available metabolic profiles in UKB were omega-3 supplementation and/or lipid lowering drug users. To address this, we recalculated the associations between the genetic variants used as instruments in the analysis and the 8 fatty acid measurements and observed a percentage change in the effect size of the genetic associations smaller than 1% for most variants and smaller than 10% for all, indicating that our findings are not biased by the inclusion of medication or supplementation users.

## Conclusions

We have systematically investigated the phenotypic consequences of different fatty acids. We present evidence for a potentially protective effect of omega-3 fatty acid levels and DHA in particular on cholelithiasis, highlighting the need to assess them in clinical trials. At the same time, our findings do not support the supplementation of unsaturated fatty acids for CVD prevention. We found evidence for positive associations of omega-6 fatty acids and LA in particular with a range of CVD outcomes further enhancing the conflicting evidence based on fatty acids supplementation for CVD prevention and treatment.

## Supporting information

S1 STROBE Checklist STROBE Checklist. (DOCX)

S1 Table **(a)** Descriptive characteristics of the UK Biobank samples used in the discovery phase of the study. UK Biobank participants were split into two datasets: the base dataset, where the fatty acid measurements were obtained, and the target dataset, which was used to extract information on the clinical diagnoses (outcome data). **(b)** Descriptive characteristics of the independent cohorts used in the replication phase.
(XLSX)

S2 Table Phenotypic and genetic correlation matrices between circulating fatty acid measurements in 117,143 UK Biobank samples. Genetic correlations were estimated using LD score regression.
(XLSX)

S3 Table **(a)** List of independent genetic variants used as instruments per exposure in the MR-PheWAS analysis. **(b)** Gene annotation of the genetic variants used as instruments in the MR-PheWAS analysis.
(XLSX)

S4 Table F-statistic of the associations between genetic variants and fatty acids. (XLSX)

S5 Table Number of health outcomes and cases per disease group included in the analysis. Health outcomes with more than 200 cases were considered.
(XLSX)

S6 Table Phenome-wide association study results of docosahexaenoic acid (DHA) genetic risk score. (XLSX)

S7 Table Phenome-wide association study results of linoleic acid (LA) genetic risk score. (XLSX)

S8 Table Phenome-wide association study results of monounsaturated fatty acids (MUFAs) genetic risk score. (XLSX)

S9 Table Phenome-wide association study results of omega-3 fatty acids genetic risk score. (XLSX)

S10 Table Phenome-wide association study results of omega-6 fatty acids genetic risk score. (XLSX)

S11 Table Phenome-wide association study results of polyunsaturated fatty acids (PUFAs) genetic risk score. (XLSX)

S12 Table Phenome-wide association study results of saturated fatty acids (SFAs) genetic risk score. (XLSX)

S13 Table Phenome-wide association study results of total fatty acids genetic risk score. (XLSX)

S14 Table Mendelian randomisation results for the effect of fatty acids on health outcomes. 114 associations were taken forward for MR analysis. 60 associations were statistically significant (*P* < 0.05) in MR-IVW, MR-weighted median and MR-PRESSO methods, with consistent MR-Egger estimate in the same direction as MR-IVW and nonsignificant MR-Egger intercept.
(XLSX)

S15 Table Two-sample mendelian randomisation in the replication phase using Kettunen and colleagues’ betas for the fatty acids and FinnGen, CARDIoGRAMplusC4D, GIANT, MAGIC, IGAP, and DIAGRAM summary statistics for health outcomes with available summary statistics. (XLSX)

S16 Table Two-sample mendelian randomisation results for CHD, cholelithiasis, and BMI, after excluding genetic variants from the FADS locus. (XLSX)

S17 Table Two-sample mendelian randomisation in the discovery phase using UKB for the fatty acids and CARDIoGRAMplusC4D summary statistics for coronary heart disease. (XLSX)

S18 Table Multivariable mendelian randomisation and mendelian randomisation–Bayesian method averaging (MR-BMA) results for the direct effect of fatty acids on coronary heart disease using summary statistics from CARDIoGRAMplusC4D. Both methods were conducted on three separate models. Model 1: DHA, LA, MUFA, SFA; Model 2: omega-3, omega-6, MUFA, SFA; Model 3: PUFA, MUFA, SFA; Model 4: DHA, LA; Model 5: omega-3, omega-6.
(XLSX)

S1 Fig PheWAS Manhattan plot. 845 clinical diagnoses were regressed against docosahexaenoic acid (DHA) genetic risk score (GRS). Age, sex, and the first 10 genetic principal components were used as covariates in the logistic regressions.
(DOCX)

S2 Fig PheWAS Manhattan plot. 845 clinical diagnoses were regressed against linoleic acid (LA) genetic risk score (GRS). Age, sex, and the first 10 genetic principal components were used as covariates in the logistic regressions.
(DOCX)

S3 Fig PheWAS Manhattan plot. 845 clinical diagnoses were regressed against monounsaturated fatty acids (MUFA) genetic risk score (GRS). Age, sex, and the first 10 genetic principal components were used as covariates in the logistic regressions.
(DOCX)

S4 Fig PheWAS Manhattan plot. 845 clinical diagnoses were regressed against omega-3 fatty acids genetic risk score (GRS). Age, sex, and the first 10 genetic principal components were used as covariates in the logistic regressions.
(DOCX)

S5 Fig PheWAS Manhattan plot. 845 clinical diagnoses were regressed against omega-6 fatty acids genetic risk score (GRS). Age, sex, and the first 10 genetic principal components were used as covariates in the logistic regressions.
(DOCX)

S6 Fig PheWAS Manhattan plot. 845 clinical diagnoses were regressed against polyunsaturated fatty acids (PUFA) genetic risk score (GRS). Age, sex, and the first 10 genetic principal components were used as covariates in the logistic regressions.
(DOCX)

S7 Fig PheWAS Manhattan plot. 845 clinical diagnoses were regressed against saturated fatty acids (SFA) genetic risk score (GRS). Age, sex, and the first 10 genetic principal components were used as covariates in the logistic regressions.
(DOCX)

S8 Fig PheWAS Manhattan plot. 845 clinical diagnoses were regressed against total fatty acids genetic risk score (GRS). Age, sex, and the first 10 genetic principal components were used as covariates in the logistic regressions.
(DOCX)

## Abbreviations

BMI: body mass index
CHD: coronary heart disease
CVD: cardiovascular disease
DHA: docosahexaenoic acid
EPA: eicosapentaenoic acid
FA: fatty acid
FDR: false discovery rate
GRS: genetic risk score
GWAS: genome-wide association study
HES: Hospital Episode Statistics
HWE: Hardy–Weinberg equilibrium
IVW: inverse-variance weighted
LA: linoleic acid
LD: linkage disequilibrium
LDSC: linkage disequilibrium score regression
MAF: minor allele frequency
MR: mendelian randomization
MR-BMA, MR: Bayesian method averaging
MUFA: monounsaturated fatty acid
NMR: nuclear magnetic resonance
OR: odds ratio
PheWAS: phenome-wide association study
PUFA: polyunsaturated fatty acid
RCT: randomised controlled trial
SFA: saturated fatty acid
SNP: single nucleotide polymorphism
T2D: type 2 diabetes
UKB: UK Biobank
