# Supplementary material for: Associations of genetically predicted fatty acid levels across the phenome: A mendelian randomisation study
Source: PLoS Med. 2022 Dec 29;19(12):e1004141. doi: 10.1371/journal.pmed.1004141 (PMC9799317; doi:10.1371/journal.pmed.1004141)
Supplement: S1 STROBE Checklist — (DOCX) [file pmed.1004141.s001.docx]

**STROBE-MR checklist of recommended items to address in reports of Mendelian randomization studies**^1^ ^2^

| **Item No.** | **Section** | **Checklist item** | **Page No.** | **Relevant text from manuscript** |
| --- | --- | --- | --- | --- |
| 1 | **TITLE and ABSTRACT** | Indicate Mendelian randomization (MR) as the study’s design in the title and/or the abstract if that is a main purpose of the study | Title and abstract | Associations of genetically predicted fatty acid levels across the phenome: a Mendelian randomisation study |
|  | **INTRODUCTION** |  |  |  |
| 2 | **Background** | Explain the scientific background and rationale for the reported study. What is the exposure? Is a potential causal relationship between exposure and outcome plausible? Justify why MR is a helpful method to address the study question | Introduction par 1 and 2 | Fatty acids (FA) are major constituents of several lipid species and are involved in diverse metabolic pathways and biochemical processes in normal cells [1]. Their role in health and disease has been extensively studied, while fatty acid supplements are the most commonly consumed nonvitamin/nonmineral dietary supplements by both adults and children in western societies [2] [3]. Nonetheless, the mechanisms by which fatty acids may exert their effects on health outcomes are still not fully understood and the causality between different fatty acids and health outcomes has not been established. Numerous epidemiological studies have investigated the quantity and quality of dietary fatty acids in relation to several chronic diseases including cardiovascular disease (CVD), cancers, neurodegenerative conditions and inflammatory diseases [4] [5]. However, the evidence remains inconclusive and it is often not supported by Randomized Controlled Trials (RCTs) on fatty acid supplementation [6] [7] [8] [9] [10]. The human fatty acid metabolome partly reflects the dietary fatty acid intake and has the potential to explain their pathophysiological roles. |
| 3 | **Objectives** | State specific objectives clearly, including pre-specified causal hypotheses (if any). State that MR is a method that, under specific assumptions, intends to estimate causal effects | Introduction par 2 | The present study aimed to assess potentially causal associations between circulating fatty acid levels and the risk of different health outcomes using Mendelian randomisation (MR) analysis. MR analysis uses genetic polymorphisms that are randomised by nature and can overcome the limitations of traditional epidemiological studies, as the random allocation of alleles during conception minimises confounding, and their presence from birth prevents reverse causation. Our study leveraged recent genetic data generated from over 115,000 UK Biobank (UKB) participants on circulating levels of 8 nuclear magnetic resonance (NMR) fatty acid measures, providing unprecedented power for fatty acids MR studies. Specifically, we investigated associations for docosahexaenoic acid and omega-3 fatty acids, linoleic acid and omega-6 fatty acids, as well as the fatty acid classes: monounsaturated fatty acids, polyunsaturated fatty acids, saturated fatty acids and total fatty acids. We implemented a systematic approach to examine causal pathways between each fatty acid trait and the human phenome, followed by multivariable MR and the Mendelian randomisation Bayesian model averaging (MR-BMA) framework to identify direct effects of different fatty acids and to select the ones that are the most likely causal risk factors for disease phenotypes. |
|  | **METHODS** |  |  |  |
| 4 | **Study design and data sources** | Present key elements of the study design early in the article. Consider including a table listing sources of data for all phases of the study. For each data source contributing to the analysis, describe the following: |  |  |
|  | a) | Setting: Describe the study design and the underlying population, if possible. Describe the setting, locations, and relevant dates, including periods of recruitment, exposure, follow-up, and data collection, when available. | Study design, data sources: UK biobank sections | The UK Biobank [11] is a large ongoing prospective study involving over 500,000 participants aged 40 to 69 years at recruitment from 2006 to 2010. The study has collected biological samples and a wide range of phenotypic data from its participants, including data from questionnaires, physical measures, sample assays, genome-wide genotyping and longitudinal follow-up for a plethora of health-related outcomes. Details of genotyping and data management have been described |
|  | b) | Participants: Give the eligibility criteria, and the sources and methods of selection of participants. Report the sample size, and whether any power or sample size calculations were carried out prior to the main analysis | Statistical analysis section: Genetic risk score generation subsection | For the GRS generation, we excluded non-European participants, one randomly selected participant from each pair of up to third degree relatives (kinship coefficient > 0.0884) [12] and individuals with discordant reported sex and genetic sex. Fatty acid GRS was calculated on UKB participants, after excluding those with available NMR metabolic biomarker data, so as to avoid sample overlap between the base dataset (the 117143 UKB samples used for the estimation of the effect of SNPs on the exposures) and the target dataset (the 322232 UKB samples used for the generation of the GRS, after excluding the base dataset). |
|  | c) | Describe measurement, quality control and selection of genetic variants | Genetic instruments section | Single nucleotide polymorphisms (SNPs) with a minor allele frequency (MAF) greater than 0.1 and a Hardy-Weinberg equilibrium (HWE) P-value greater than 10-6 were considered. Multi-allelic SNPs were excluded from the analysis. Independent SNPs associated with each of the 8 fatty acid concentrations were identified at a genome-wide significance threshold (P<5x10-8). Independence of SNPs was assessed through clumping for linkage disequilibrium (LD) r2<0.001 using a window of 10 million bases and the 1000 genomes phase 3 European reference panel through the open-source association toolset plink |
|  | d) | For each exposure, outcome, and other relevant variables, describe methods of assessment and diagnostic criteria for diseases | Data sources: Health outcomes subsection | Individual-level data from UKB were used for clinical health outcomes with more than 200 cases, obtained from national registries, such as Hospital Episode Statistics (HES), Cancer registry and death registry. Cases were defined according to ICD-9 and ICD-10 codes (international classification of diseases, 9th and 10th revisions) |
|  | e) | Provide details of ethics committee approval and participant informed consent, if relevant |  |  |
| 5 | **Assumptions** | Explicitly state the three core IV assumptions for the main analysis (relevance, independence and exclusion restriction) as well assumptions for any additional or sensitivity analysis | Statistical analysis: Two-sample Mendelian randomisation and sensitivity analyses subsection | Mendelian randomisation assesses causal relationships using genetic variants as instrumental variables. A genetic variant can be considered as an instrumental variable if it satisfies three assumptions: it is robustly associated with the exposure, it is independent of any confounders of the exposure – outcome relationship and is associated with the outcome only via the exposure. |
| 6 | **Statistical methods: main analysis** | Describe statistical methods and statistics used |  |  |
|  | a) | Describe how quantitative variables were handled in the analyses (i.e., scale, units, model) | Statistical analysis: Two-sample Mendelian randomisation and sensitivity analyses subsection | All estimates were reported as odds ratio (OR) per one mmol/L increase in fatty acid concentrations, together with their 95% confidence interval. |
|  | b) | Describe how genetic variants were handled in the analyses and, if applicable, how their weights were selected | Genetic instruments section | Single nucleotide polymorphisms (SNPs) with a minor allele frequency (MAF) greater than 0.1 and a Hardy-Weinberg equilibrium (HWE) P-value greater than 10-6 were considered. Multi-allelic SNPs were excluded from the analysis. Independent SNPs associated with each of the 8 fatty acid concentrations were identified at a genome-wide significance threshold (P<5x10-8). Independence of SNPs was assessed through clumping for linkage disequilibrium (LD) r2<0.001 using a window of 10 million bases and the 1000 genomes phase 3 European reference panel through the open-source association toolset plink [20]. |
|  | c) | Describe the MR estimator (e.g. two-stage least squares, Wald ratio) and related statistics. Detail the included covariates and, in case of two-sample MR, whether the same covariate set was used for adjustment in the two samples | Statistical analysis: Two-sample Mendelian randomisation and sensitivity analyses subsection | The main analysis was conducted using the random-effects inverse variance weighted (IVW) method, which provides precise causal estimates, assuming that all variants are valid instrumental variables [22]. In sensitivity analyses, we applied the MR-Egger method to explore the assumption of no pleiotropy in genetic instruments [23]. In addition, we performed the MR-weighted median method, which returns an accurate causal estimate, provided that at least 50% of the weight in the analysis comes from valid instrumental variables [24]. The I2 statistic was calculated to detect heterogeneity among the MR estimates obtained from multiple genetic variants. Last, we used MR-PRESSO, which detects and removes pleiotropic variants based on their contributions to heterogeneity [25]. |
|  | d) | Explain how missing data were addressed | Not applicable | Not applicable |
|  | e) | If applicable, indicate how multiple testing was addressed | Results: Phenome-wide association study section | Thirty-five unique clinical outcomes were associated with at least one fatty acid GRS at 5% FDR threshold (P=9.2x10⁻⁴). |
| 7 | **Assessment of assumptions** | Describe any methods or prior knowledge used to assess the assumptions or justify their validity | Statistical analysis: Two-sample Mendelian randomisation and sensitivity analyses subsection | In sensitivity analyses, we applied the MR-Egger method to explore the assumption of no pleiotropy in genetic instruments [23]. In addition, we performed the MR-weighted median method, which returns an accurate causal estimate, provided that at least 50% of the weight in the analysis comes from valid instrumental variables [24]. The I2 statistic was calculated to detect heterogeneity among the MR estimates obtained from multiple genetic variants. Last, we used MR-PRESSO, which detects and removes pleiotropic variants based on their contributions to heterogeneity [25]. […] The F-statistic for each genetic instrument was generated to assess their strength. We included instruments with F-statistic > 10 in the analysis. |
| 8 | **Sensitivity analyses and additional analyses** | Describe any sensitivity analyses or additional analyses performed (e.g. comparison of effect estimates from different approaches, independent replication, bias analytic techniques, validation of instruments, simulations) | Data sources: Health outcomes subsection | In the replication phase we used the Kettunen et al. GWAS [14] as an alternative source of beta estimates for the associations between the genetic variants and circulating fatty acids. We also used the GIANT consortium for body mass index (BMI) [15], CARDIoGRAMplusC4D for coronary heart disease (CHD) [16] and FinnGen (https://r5.finngen.fi/) for cholelithiasis and “other biliary tract disease”. Additionally, we used the largest available GWAS summary statistics for diseases which there has been evidence of association with fatty acids in the literature, namely, IGAP [17] for Alzheimer’s disease, MAGIC [18] for HbA1c and DIAGRAM [19] for type 2 diabetes (T2D). |
| 9 | **Software and pre-registration** |  |  |  |
|  | a) | Name statistical software and package(s), including version and settings used | Statistical software subsection | Analysis was conducted in R version 4.0.2 [29], phenome-wide associations were conducted using R package “PheWAS” [30], two-sample MR was performed using “TwoSampleMR” [31] and “MRPRESSO” [25] R packages, MR-BMA was conducted using the methodology provided in [27]. Figures were produced using the R package “forestplot” [32]. |
|  | b) | State whether the study protocol and details were pre-registered (as well as when and where) |  |  |
|  | **RESULTS** |  |  |  |
| 10 | **Descriptive data** |  |  |  |
|  | a) | Report the numbers of individuals at each stage of included studies and reasons for exclusion. Consider use of a flow diagram | Results par 1 | Descriptive characteristics of the base and target datasets used in the discovery phase and the cohorts used in the replication phase are provided in Supplementary Table 1. |
|  | b) | Report summary statistics for phenotypic exposure(s), outcome(s), and other relevant variables (e.g. means, SDs, proportions) | Results par 1 | Descriptive characteristics of the base and target datasets used in the discovery phase and the cohorts used in the replication phase are provided in Supplementary Table 1. |
|  | c) | If the data sources include meta-analyses of previous studies, provide the assessments of heterogeneity across these studies |  |  |
|  | d) | For two-sample MR:  i.  Provide justification of the similarity of the genetic variant-exposure associations between the exposure and outcome samples  ii.  Provide information on the number of individuals who overlap between the exposure and outcome studies | i)Methods: Statistical analysis section, Genetic risk score generation subsection  ii) Methods: Two-sample Mendelian randomisation and sensitivity analyses section | i) Fatty acid GRS was calculated on UKB participants, after excluding those with available NMR metabolic biomarker data, so as to avoid sample overlap between the base dataset (the 117143 UKB samples used for the estimation of the effect of SNPs on the exposures) and the target dataset (the 322232 UKB samples used for the generation of the GRS, after excluding the base dataset).  ii) In this MR framework there was no sample overlap between the UKB populations used for the fatty acids (base dataset) and for the clinical diagnoses (target dataset). |
| 11 | **Main results** |  |  |  |
|  | a) | Report the associations between genetic variant and exposure, and between genetic variant and outcome, preferably on an interpretable scale | Results par 1 | The hierarchical order of the fatty acid measurements, phenotypic and genetic correlations between the fatty acids and the genetic variants used as instruments in the analysis are provided in Figure 2. |
|  | b) | Report MR estimates of the relationship between exposure and outcome, and the measures of uncertainty from the MR analysis, on an interpretable scale, such as odds ratio or relative risk per SD difference | Results: Mendelian randomisation section, par 1-4 |  |
|  | c) | If relevant, consider translating estimates of relative risk into absolute risk for a meaningful time period |  |  |
|  | d) | Consider plots to visualize results (e.g. forest plot, scatterplot of associations between genetic variants and outcome versus between genetic variants and exposure) | Results: Mendelian randomisation section, par 1-4 |  |
| 12 | **Assessment of assumptions** |  |  |  |
|  | a) | Report the assessment of the validity of the assumptions | Results par 1 | The list of independent genetic variants used as instrumental variables in this analysis and their corresponding F-statistics are provided in Supplementary Tables 3, 4. |
|  | b) | Report any additional statistics (e.g., assessments of heterogeneity across genetic variants, such as *I^2^*, Q statistic or E-value) | Results: Mendelian randomisation section, par 3 | Considerable heterogeneity was observed in these analyses (I2 statistic> 50%), which when corrected by removing outliers from MR-PRESSO, resulted in effect estimates supporting a causal effect. |
| 13 | **Sensitivity analyses and additional analyses** |  |  |  |
|  | a) | Report any sensitivity analyses to assess the robustness of the main results to violations of the assumptions | Results: Mendelian randomisation section, par 1-4 |  |
|  | b) | Report results from other sensitivity analyses or additional analyses | Results: Mendelian randomisation section, par 1-4 |  |
|  | c) | Report any assessment of direction of causal relationship (e.g., bidirectional MR) |  |  |
|  | d) | When relevant, report and compare with estimates from non-MR analyses |  |  |
|  | e) | Consider additional plots to visualize results (e.g., leave-one-out analyses) |  |  |
|  | **DISCUSSION** |  |  |  |
| 14 | **Key results** | Summarize key results with reference to study objectives | Discussion par 1 | In this study, we used MR to explore and compare the relationships of fatty acids levels with a wide range of clinical phenotypes across the phenome. Our principal findings include the positive associations between several genetically predicted fatty acids and CVD outcomes with multivariable analysis highlighting omega-6 fatty acids and LA in particular, having an independent causal positive effect on CHD. This adds further evidence against the supplemental use of fatty acids for CVD prevention. Our results also indicate negative associations of higher DHA and omega-3 fatty acid levels with lower risk of cholelithiasis and obesity. |
| 15 | **Limitations** | Discuss limitations of the study, taking into account the validity of the IV assumptions, other sources of potential bias, and imprecision. Discuss both direction and magnitude of any potential bias and any efforts to address them | Discussion: Strengths and limitations section | However, our results must be interpreted in the context of a few limitations. Our work was confined to individuals of European descent, thus limiting the amenability of our findings to other ethnic groups. Also, we were unable to assess the effect of fatty acid ratios such as omega-6-to-omega-3 fatty acid that might be important for some of the examined clinical outcomes. Although our study strengthens the case for a causal relationship between fatty acids and several diseases, we cannot prove causality and our findings require further evaluation in experimental studies and RCTs. For example, pleiotropy is always a potential problem in MR and occurs when a genetic instrument is associated with the outcome through pathways not including the exposure of interest. We used extensive sensitivity analyses to account for pleiotropy and implemented a Bayesian multivariable approach to select among the studied fatty acids the one more likely to be causal. This is important as the examined fatty acids have high genetic correlation. Therefore, the observed associations could reflect an association between a fatty acid other than those we have investigated, or even a combination of fatty acids. Lastly, exogenous factors may affect the genetic associations with the fatty acids, since 13577 participants with available metabolic profiles in UKB were omega-3 supplementation and/or lipid lowering drug users. To address this, we recalculated the associations between the genetic variants used as instruments in the analysis and the eight fatty acid measurements and observed a percentage change in the effect size of the genetic associations smaller than 1% for most variants and smaller than 10% for all, indicating that our findings are not biased by the inclusion of medication or supplementation users. |
| 16 | **Interpretation** |  |  |  |
|  | a) | Meaning: Give a cautious overall interpretation of results in the context of their limitations and in comparison with other studies | Discussion par 1-3 |  |
|  | b) | Mechanism: Discuss underlying biological mechanisms that could drive a potential causal relationship between the investigated exposure and the outcome, and whether the gene-environment equivalence assumption is reasonable. Use causal language carefully, clarifying that IV estimates may provide causal effects only under certain assumptions | Discussion par 1-3 |  |
|  | c) | Clinical relevance: Discuss whether the results have clinical or public policy relevance, and to what extent they inform effect sizes of possible interventions | Discussion par 1-3 |  |
| 17 | **Generalizability** | Discuss the generalizability of the study results (a) to other populations, (b) across other exposure periods/timings, and (c) across other levels of exposure | Discussion: Strengths and limitations section | Our work was confined to individuals of European descent, thus limiting the amenability of our findings to other ethnic groups. Also, we were unable to assess the effect of fatty acid ratios such as omega-6-to-omega-3 fatty acid that might be important for some of the examined clinical outcomes. |
|  | **OTHER INFORMATION** |  |  |  |
| 18 | **Funding** | Describe sources of funding and the role of funders in the present study and, if applicable, sources of funding for the databases and original study or studies on which the present study is based | Funding section | This research has been conducted using the UK Biobank Resource under application 236 The funders were not involved in the analysis and interpretation of the data; in the writing of the report; or in the decision to submit the paper for publication. |
| 19 | **Data and data sharing** | Provide the data used to perform all analyses or report where and how the data can be accessed, and reference these sources in the article. Provide the statistical code needed to reproduce the results in the article, or report whether the code is publicly accessible and if so, where | Data access section | UK Biobank individual level data used in this work can be accessed after applying for access at (https://www.ukbiobank.ac.uk/enable-your-research/apply-for-access). Summary statistics used in the analysis can be openly accessed at the IEU OpenGWAS project (https://gwas.mrcieu.ac.uk/). |
| 20 | **Conflicts of Interest** | All authors should declare all potential conflicts of interest | Competing interests section | DG is employed part-time by Novo Nordisk. VZ is a paid statistical consultant on PLOS Medicine's statistical board. |

This checklist is copyrighted by the Equator Network under the Creative Commons Attribution 3.0 Unported (CC BY 3.0) license.

1. Skrivankova VW, Richmond RC, Woolf BAR, Yarmolinsky J, Davies NM, Swanson SA, et al. Strengthening the Reporting of Observational Studies in Epidemiology using Mendelian Randomization (STROBE-MR) Statement. JAMA. 2021;under review.

2. Skrivankova VW, Richmond RC, Woolf BAR, Davies NM, Swanson SA, VanderWeele TJ, et al. Strengthening the Reporting of Observational Studies in Epidemiology using Mendelian Randomisation (STROBE-MR): Explanation and Elaboration. BMJ. 2021;375:n2233.
