# Supplementary material for: Associations of genetically predicted fatty acid levels across the phenome: A mendelian randomisation study
Source: PLoS Med. 2022 Dec 29;19(12):e1004141. doi: 10.1371/journal.pmed.1004141 (PMC9799317; doi:10.1371/journal.pmed.1004141)
Supplement: S4 Fig — 845 clinical diagnoses were regressed against omega-3 fatty acids genetic risk score (GRS). Age, sex, and the first 10 genetic principal components were used as covariates in the logistic regressions. (DOCX) [file pmed.1004141.s023.docx]

**Supplementary Figure 4**. PheWAS Manhattan plot. 845 clinical diagnoses were regressed against omega-3 fatty acids genetic risk score (GRS). Age, sex and the first 10 genetic principal components were used as covariates in the logistic regressions.
