# Supplementary material for: Associations of genetically predicted fatty acid levels across the phenome: A mendelian randomisation study
Source: PLoS Med. 2022 Dec 29;19(12):e1004141. doi: 10.1371/journal.pmed.1004141 (PMC9799317; doi:10.1371/journal.pmed.1004141)
Supplement: S6 Fig — 845 clinical diagnoses were regressed against polyunsaturated fatty acids (PUFA) genetic risk score (GRS). Age, sex, and the first 10 genetic principal components were used as covariates in the logistic regressions. (DOCX) [file pmed.1004141.s025.docx]

**Supplementary Figure 6**. PheWAS Manhattan plot. 845 clinical diagnoses were regressed against polyunsaturated fatty acids (PUFA) genetic risk score (GRS). Age, sex and the first 10 genetic principal components were used as covariates in the logistic regressions.
